# HIF-1 transcription activity: HIF1A driven response in normoxia and in hypoxia

**DOI:** 10.1186/s12881-019-0767-1

**Published:** 2019-02-26

**Authors:** Flora Cimmino, Marianna Avitabile, Vito Alessandro Lasorsa, Annalaura Montella, Lucia Pezone, Sueva Cantalupo, Feliciano Visconte, Maria Valeria Corrias, Achille Iolascon, Mario Capasso

**Affiliations:** 1 Department of Molecular Medicine and Medical Biotechnology, University of Naples Federico II, Naples, Italy; 2 CEINGE Biotecnologie Avanzate, Naples, Italy; 3 Experimental therapy in Oncology, Istituto Giannina Gaslini, Genoa, Italy; 4 IRCCS SDN, Naples, Italy

**Keywords:** Hypoxia inducible factor HIF1A, RNA sequencing, DNA methylation, Neuroblastoma

## Abstract

**Background:**

HIF1A (Hypoxia-Inducible-Factor 1A) expression in solid tumors is relevant to establish resistance to therapeutic approaches. The use of compounds direct against hypoxia signaling and HIF1A does not show clinical efficiency because of changeable oxygen concentrations in solid tumor areas. The identification of HIF1A targets expressed in both normoxia and hypoxia and of HIF1A/hypoxia signatures might meliorate the prognostic stratification and therapeutic successes in patients with high-risk solid tumors.

**Methods:**

In this study, we conducted a combined analysis of RNA expression and DNA methylation of neuroblastoma cells silenced or unsilenced for HIF1A expression, grown in normoxia and hypoxia conditions.

**Results:**

The analysis of pathways highlights HIF-1 (heterodimeric transcription factor 1) activity in normoxia in metabolic process and HIF-1 activity in hypoxia in neuronal differentiation process. HIF1A driven transcriptional response in hypoxia depends on epigenetic control at DNA methylation status of gene regulatory regions. Furthermore, low oxygen levels generate HIF1A-dependent or HIF1A-independent signatures, able to stratify patients according to risk categories.

**Conclusions:**

These findings may help to understand the molecular mechanisms by which low oxygen levels reshape gene signatures and provide new direction for hypoxia targeting in solid tumor.

**Electronic supplementary material:**

The online version of this article (10.1186/s12881-019-0767-1) contains supplementary material, which is available to authorized users.

## Background

Neuroblastoma (NB) is a pediatric tumor derived from the sympathoadrenal lineage of neural crest progenitor cells and represents the most common malignancy in early childhood [1]. DNA and RNA aberrant profiles have been shown to identify mechanisms behind the clinical outcome of NB as the expression of several genes involved in proliferation, differentiation and metastasis that negatively impact on therapy success. Despite recent improvements in survival in randomized trials, nearly 50% of children with high-risk disease is refractory to therapy or suffer a relapse [2–4]. High-risk tumors are characterized by un-differentiated phenotype, age at diagnosis ≥18 months and harbor a very low rate of recurrent somatic mutations in both nuclear and mitochondrial DNA [5–9].

Hypoxia is an important factor in the pathology of many human diseases, including cancer, diabetes, aging, and stroke/ischemia. Low oxygen levels represent an important microenvironment condition to affect the activation status of signaling pathways as drug resistance mechanism. Indeed the increased expression of Hypoxia-Inducible-Factor HIF-1α mRNA (HIF1A) in tumors is relevant to establish resistance to therapeutic approaches as radiotherapy [10, 11]. We have recently reported that high HIF1A expression may stratify high-risk NB patients with poorer prognosis and low HIF1A expression enhances neuronal differentiation signaling pathways activation and response to differentiating agents [12]. The identification of factors able to influence the expression levels of HIF1A could allow greater therapeutic success. Recent reports suggest that HIF1-α protein might be degraded in VHL-independent manner following intracellular accumulation of methylglioxal (MGO), a highly reactive α-oxoaldehyde formed as a by-product of glycolisis [13, 14]. Polymorphisms in glucoxylase I enzyme (GLOI) results in down-regulation of GLOI enzyme that play important role in MGO detoxyfication and favor damage from oxydative stress and the degradation pathway of HIF1A [15]. Indeed, conditions with increased availability of glucose, such as diabetes or down-regulation of GLOI highlight the importance of mechanisms to disrupt cell response to hypoxia.

Tumor cells respond to repeated oxygen levels fluctuations in tumor microenvironment through epigenetic control. Epigenetic regulatory mechanisms are coordinated at several levels: i) DNA, by (hydroxy) methylation of CpG islands (CGI), ii) RNA, through involvement of regulatory noncoding RNA, and iii) proteins, by activation of epigenetic regulators and posttranslational modificators of histones. Their concerted action in hypoxia drives tumor plasticity through the acquisition of local or global chromatin modifications, which allow the accessibility of hypoxia-responsive elements (HRE) loci or of new active DNA regions at hypoxia inducible factors [16].

Epigenetic regulation of gene expression by DNA methylation plays a central role in determining tissue specific gene expression and chromosome instability. In cancer, the DNA methylation landscape is very complex: promoter CGIs hypermethylation is associated to inactivation of tumor suppressors as well as the presence of DNA hypomethylation blocks and contiguously hypermethylated CGIs at telomeric regions [17, 18]. Several studies show HIF1A expression can control DNA hypomethylation status of HRE. Interestingly, more than half of histone demethylase belonged to Jumonji C family genes were up-regulated by hypoxia and four of them (JMJD1A, JMJD2B, JMJD2C, PLU-1) were reported to be direct HIF1A targets and may result in increased HIF-1α binding to the HRE [19, 20].

Tumor hypoxia acts as a novel regulator of DNA methylation independently of HIF1A activity. High levels of hypoxia metabolites as succinate and fumarate altered the global DNA methylation patterns via significant DNA hypermethylation [21]. Activity of ten-eleven translocation (TET) enzymes that catalyze DNA demethylation through 5-methylcytosine oxidation depends directly on oxygen shortage. Indeed, TETs activity is reduced by tumor hypoxia in human and mouse cells [22]. Although HIF1A plays a role in defining DNA methylation status of its targets, its role in the global hypermethylation induced by hypoxia remains to be explored [23].

To shed light on the molecular mechanisms by which hypoxia reshapes gene expressions of tumors, we have performed an integrated analysis of gene expression and DNA methylation in NB cells upon HIF1A inhibition in normoxia and hypoxia conditions. We found that HIF1A transcription response in hypoxia is driven by epigenetic control of low oxygen levels and can upgrade high-risk tumor features. Interestingly, HIF1A targets expressed in both normoxic and hypoxic areas may provide novel targets to eradicate solid tumors.

## Methods

### Cell culture

The human SKNBE2 (ATCC #CRL-2271) cell line was grown in Dulbecco’s modified Eagle’s medium supplemented with 10% heat inactivated fetal bovine serum (Sigma), 1 mM L-glutamine, penicillin (100 U/ml) and streptomycin (100 μ g/ml) (Invitrogen), at 37 °C, under 5% CO2 in a humidified atmosphere. The cells exposed to hypoxia were grown at 0.5% oxygen for 2 h. The cells used for all the experiments were re-authenticated and tested as mycoplasma-free. Early-passage cells were used and cumulative culture length was less than 3 months after resuscitation.

### Lentiviral production to knock-down HIF1A expression

To knock-down HIF1A expression, the pGIPZ lentiviral shRNAmir that targets human HIF1A were purchased from Open Biosystems (Thermo Fisher Scientific, Inc.). We used two different shRNAs against HIF1A: V2LHS_132152 (RHS4430–98513964) (shHIF1A#A) and V2LHS_236x718 (RHS443098513880) (shHIF1A#B). A non-silencing pGIPZ lentiviral shRNAmir was used as the control (RHS4346). The production of lentivirus particles and cells infection was performed as previously described [12]. To obtain 100% GFP-positive cells, puromycin was added into the medium for an additional 10 days.

### Fractionation of nuclear proteins and western blotting

Cell pellets were resuspended in a hypo-tonic buffer (10 mM HEPES-K +, pH 7.5, 10 mM KCl, 1.5 mM MgCl 2, 0.5 M dithiothreitol) in the presence of a protease inhibitors cocktail (Roche). The cells were lysed by addition of ice-cold 0.5% NP-40 for 10 min. The nuclei were pelleted at 1000 x g for 2 min at 4 °C and nuclear protein extraction and concentrations was determined as previously described [12]. Protein membranes were probed with anti-HIF-1α (610,959; BD Biosciences) and horseradish-peroxidase-conjugated anti-mouse secondary antibody (1:4000 dilution; ImmunoReagent). Positive bands were visualized using the ECL kit SuperSignal West Pico Chemiluminescent Substrate (Pierce). A rabbit anti-H3 antibody (ab1791 Abcam) was used as the control for equal loading.

### RNA isolation, cDNA library construction and sequencing

Total RNA was isolated from NB cell line using TRIzol LS Reagent (Invitrogen) according to manufacturer’s instructions; samples quality and library construction is described in Additional file 1. cDNA Sequencing was accomplished using an Illumina HiSeq™ 2000 platform according to the manufacturer’s protocols (Analysis performed at BIOGEM facility). Illumina paired end sequencing protocol yielded about 20 millions of 2x101nt reads with high quality bases (mean quality of 34) and mean % GC of 46.

### Analysis of differentially expressed genes and gene set enrichment

Sequencing data were analyzed with the set of open source programs of the Tuxedo suite: TopHat v2.0.14 (for sequence alignment) and Cufflinks v2.1.0 (for differential expression analysis), following the pipeline published in Nature Protocols by Trapnell et al., 2012 (see Additional file 1 for further details) [24]. The set of output files obtained by Cufflinks was inspected and explored using the R-Bioconductor package CummeRbund v2.16.0, which provides functions to read, subset, filter and plot results. We selected genes differentially expressed in each of the pairwise comparisons if the Benjamini Hochberg adjusted *P*-value (FDR) was under 0.05 and if the Log_2_ transformed fold change was greater than + 0.5 (up-regulated) or lower than − 0.5 (down-regulated). The lists of these genes were used to query Pathway and Gene Ontology databases. The functional enrichment analysis tool Webgestalt (WEB-based GEne SeT AnaLysis Toolkit) was used to detect significant enrichments for Kyoto Encyclopedia of Genes and Genomes (KEGG) pathways. The enrichment analysis was performed using the following criteria: an hypergeometric test for statistical analysis, FDR ≤ 0.05 and 10 as minimum number of genes for a category. Data generated during this study are included in this manuscript and in Additional files 1, 2 and 3.

### DNA extraction, bisulfite modification and DNA methylation array hibridization

DNA extraction was carried out with the Wizard Genomic DNA Purification Kit (Promega, WI, USA), including a RNA removal step, according to the protocol provided by the supplier. The DNA was quantified with the Nanodrop and 1 μg was used for bisulfite modification using EZ-96 DNA Methylation™ Kit (Zymo Research CA, USA) with the modification step according to the recommendations for array processing of the samples. Control PCRs were carried out before array analysis to confirm successful modification of the DNA. The bisulfite-modified DNA (500 ng) was laid on the Infinium HumanMethylation450 BeadChips (Illumina), which determine the methylation levels of 485,000 CpG sites. The fluorescence signals were measured from the BeadArrays using an Illumina BeadStation GX scanner. The raw fluorescence images (IDAT files) were then analyzed using R and R-Bioconductor packages. The ChAMP package was used for data preprocessing, normalization and comparison between groups [25, 26]. Singular value decomposition analysis was performed to identify confounding factors and evaluate possible batch effects, while the SWAN (Subset Within Array Normalization) method was used for probe intensity normalization. After these steps the fraction of failed probes was about 0.003%.

### Analysis of differentially methylated CpGs, CpGs enrichment and correlation to expression

ChAMP assigns a score called “β value” to each CpG site, which corresponds to the ratio between the fluorescence signal of the methylated allele (C) and the unmethylated (T) alleles. The β value, ranging from 0 to 1, represents the methylation status of each probe from totally unmethylated (β = 0) to totally methylated (β = 1). The software was used to calculate probes that were differentially methylated between groups. CpG sites were considered as differentially methylated, in a contrast, if Δβ (delta beta) was below − 0.2 (hypo-methylation) or above 0.2 (hyper-methylation) and the FDR was lower than 0.05. Data generated during this study are included in this manuscript and in Additional files 1, 2 and 3.

### Real-time RT-PCR

The expression levels of 13 genes were analyzed using real-time, quantitative PCR in SKNBE2 shHIF1A#A and shCTR cells. Total RNA extraction using TRIzol LS Reagent (Invitrogen) and cDNA retrotranscription using the High Capacity cDNA Reverse Transcription Script (Applied Biosistem) was performed according to the manufacturer protocol. The cDNA samples were diluted to 20 ng/μ l. Gene-specific primers were designed by using PRIMEREXPRESS software (Applied Biosystems) and primers sequences for each gene are listed in Additional file 1. Real-time PCR was performed using SYBR Green PCR Master Mix (AppliedBiosystems). All real-time PCR reactions were performed using the 7900HT Fast Real-Time PCR System (Applied Biosystems). The experiments were carried out in triplicate for each data point. The housekeeping gene β -actin was used as the internal control. Relative gene expression was calculated using the 2 ^−ΔΔCT^ method as described in our previous work [27], where the ∆CT was calculated using the differences in the mean CT between the selected genes and the internal control (β -actin). The mean fold change of 2 − (average ∆∆CT) was determined using the mean difference in the ∆CT between the gene of interest and the internal control.

## Results

### HIF1A driven response in normoxia and in hypoxia conditions

**Fig. 1:**
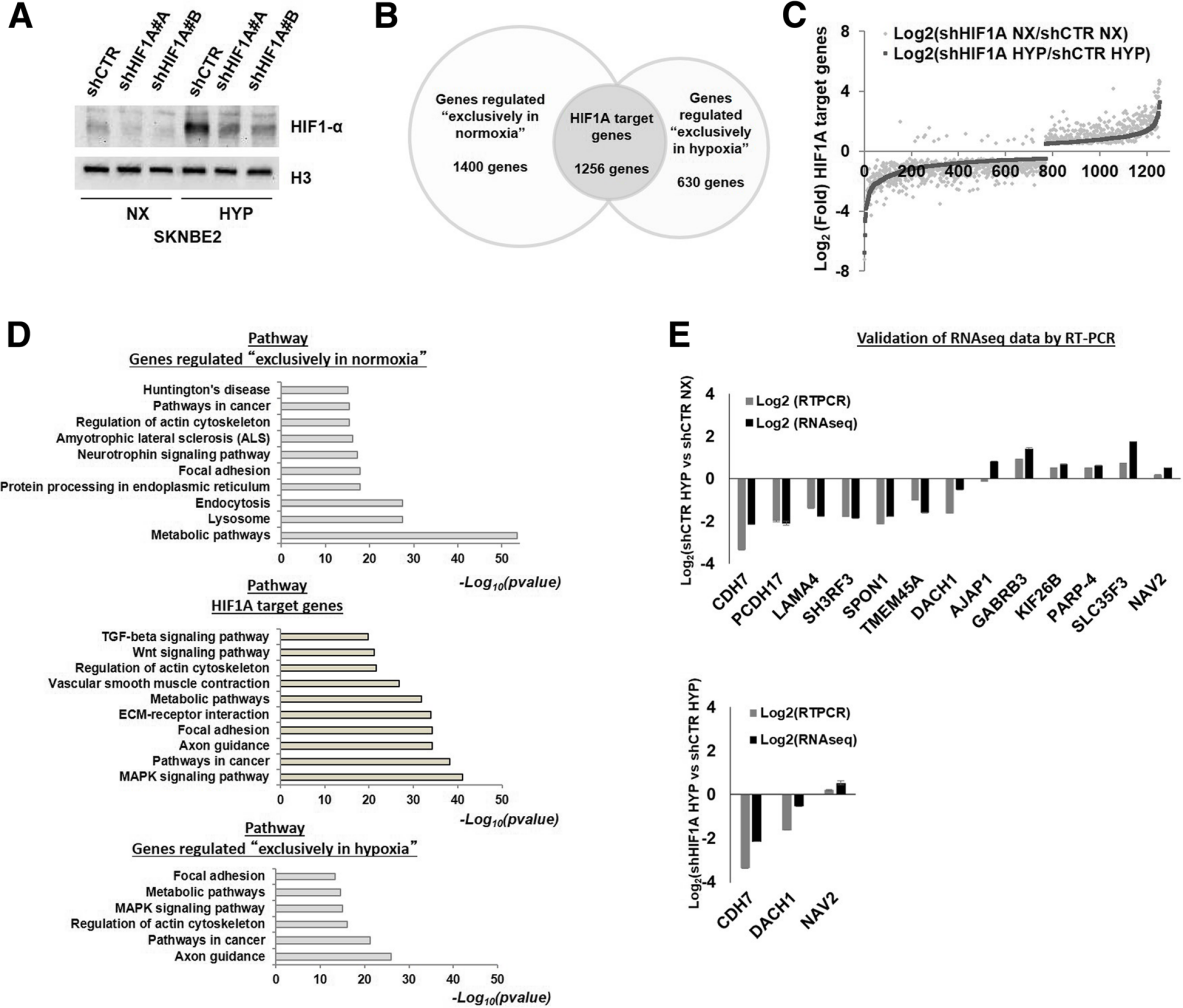
HIF1A driven response in normoxia and in hypoxia conditions. **a** HIF1A depletion in SKNBE2 was verified by western blotting. The silencing was madiated by two short hairpin against HIF1A (shHIF1A#A and shHIF1A#B). Unsilenced cells were used as control (shCTR). SKNBE2 shHIF1#B was used for RNA sequencing experiments. **b** The differentially expressed genes in shHIF1A NX vs shCTR NX and in shHIF1A HYP vs shCTR HYP gene sets were crossed and three gene lists were obtained: genes regulated “exclusively in normoxia”, genes regulated “exclusively in hypoxia” and HIF1A target genes. The number of genes for each gene list is reported in the graph. **c** The Log2 expression of HIF1A target genes is reported for each gene set. **d** KEGG pathway analysis (webGestalt) of the three gene lists is shown. The negative Log_10_ pvalue is reported on X-axis (FDR ≤ 0.05). **e** The reliability of RNAseq data was estimated by assessing the expression values of chosen genes by RT-PCR in SKNBE2 shHIF1A#A and shCTR cells. Log_2_Fold of expression in RT-PCR and RNAseq experiments is reported

SKNBE2 NB cells have biochemical features of neurons and display NMYC amplification, a marker of NB malignant progression. SKNBE2 cells were depleted for HIF1A expression (shHIF1A) by the use of two short hairpin against HIF1A (SKNBE2 shHIF1A#A and SKNBE2 shHIF1A#B) and were grown in normoxia and hypoxia conditions (NX and HYP); unsilenced cells were used as control (SKNBE2 shCTR) (Fig. 1a). To evaluate the hypoxic status of the cells after their exposure to low oxygen conditions we tested the expression of known hypoxia targets (Additional file 2: Figure S1). To provide genes and pathways differentially regulated by HIF1A triplicates of silenced (SKNBE2 shHIF1A#B NX and SKNBE2 shHIF1A#B HYP) and unsilenced (SKNBE2 shCTR NX and SKNBE2 shCTR HYP) cells were subjected to RNA-seq experiment.

To get insights into the relationships between the experimental conditions, we performed hierarchical clustering of gene-based FPKM counts. Results clearly showed two main branches of the dendrogram separating silenced and unsilenced conditions (Additional file 2: Figure S2A). Although small changes of global expression levels were observed in the four conditions (Additional file 2: Figure S2B), principal component analysis (PCA) and multi-dimensional scaling (MDS) highlighted the important role of oxygen in separating shCTR HYP and shCTR NX whereas shHIF1A NX and shHIF1A HYP showed highly similar profiles in PCA analysis (Additional file 2: Figure S2C and D).

By comparing gene expression in shHIF1A NX vs shCTR NX and in shHIF1A HYP vs shCTR HYP, we obtained two gene sets. Raw calls of differentially expressed genes were subsequently filtered by fold change (Log_2_ ≥ + 0.5 or ≤ − 0.5) and statistical significance (FDR ≤ 0.05) (Additional file 3: Tables S1 and S2). HIF1A silencing in normoxia affects the expression of much more genes (“shHIF1A NX vs shCTR NX” includes 2656 genes) than in hypoxia (“shHIF1A HYP vs shCTR HYP” includes 1886 genes) (Additional file 2: Figure S3A). KEGG pathway analysis showed that the most significantly enriched terms were “metabolic pathway” in shHIF1A NX vs shCTR NX and “axon guidance” in shHIF1A HYP vs shCTR HYP (FDR ≤ 0.05) (Additional file 2: Figure S3B).

By intersecting the above-cited “two gene sets”, we obtained three gene lists: 1) genes (*n* = 630) regulated “exclusively in hypoxia”; 2) genes (*n* = 1400) regulated “exclusively in normoxia” and 3) a list of genes (*n* = 1256) which are commonly regulated by HIF1A that we named HIF1A target genes (Fig. 1b). The expression trend of 1237 out of 1256 HIF1A target genes (98.88%) was concordant upon HIF1A depletion in NX and HYP (Fig. 1c). Conversely, 19 genes out of 1256 (less than 1.2%) had an opposite regulation in shHIF1A NX vs shCTR NX and shHIF1A HYP vs shCTR HYP, suggesting they are downstream targets of HIF1A related pathways. KEGG pathway analysis of the two “exclusive” gene sets revealed an enrichment of metabolic pathways in normoxia and an enrichment of axon guidance and pathways in cancer in hypoxia (FDR ≤ 0.05). KEGG pathway analysis of HIF1A target genes revealed an enrichment of MAPK signaling pathway, pathways in cancer and axon guidance (FDR ≤ 0.05) (Fig. 1d).

The reliability of RNA-seq data was assessed by RT-PCR in SKNBE2 shCTR and shHIF1A#A (Fig. 1e). We validated genes that have RNAseq log_2_ fold change ranging from − 2 to 2, in shCTR HYP vs shCTR NX (Additional file 3: Table S3) and shHIF1A HYP vs shCTR HYP gene list. We found that expression levels measured by RNA-Seq were consistent with those obtained by RT-PCR.

Additionally, we confirmed these results by RT-PCR in SHSY5Y NB cells that have biochemical features of neurons but do not display high-risk marker as NMYC amplification. As described in Supplementary data, SHSY5Y cells were depleted for HIF1A expression (shHIF1A) and unsilenced cells were used as control (shCTR). The gene expression levels measured by RT-PCR in SHSY5Y cells were consistent with those obtained by RNA-Seq in SKNBE2 cells (Additional file 2: Figure S4).

### Transcription activity under hypoxia exposure is both HIF1A dependent and HIF1A independent

**Fig. 2:**
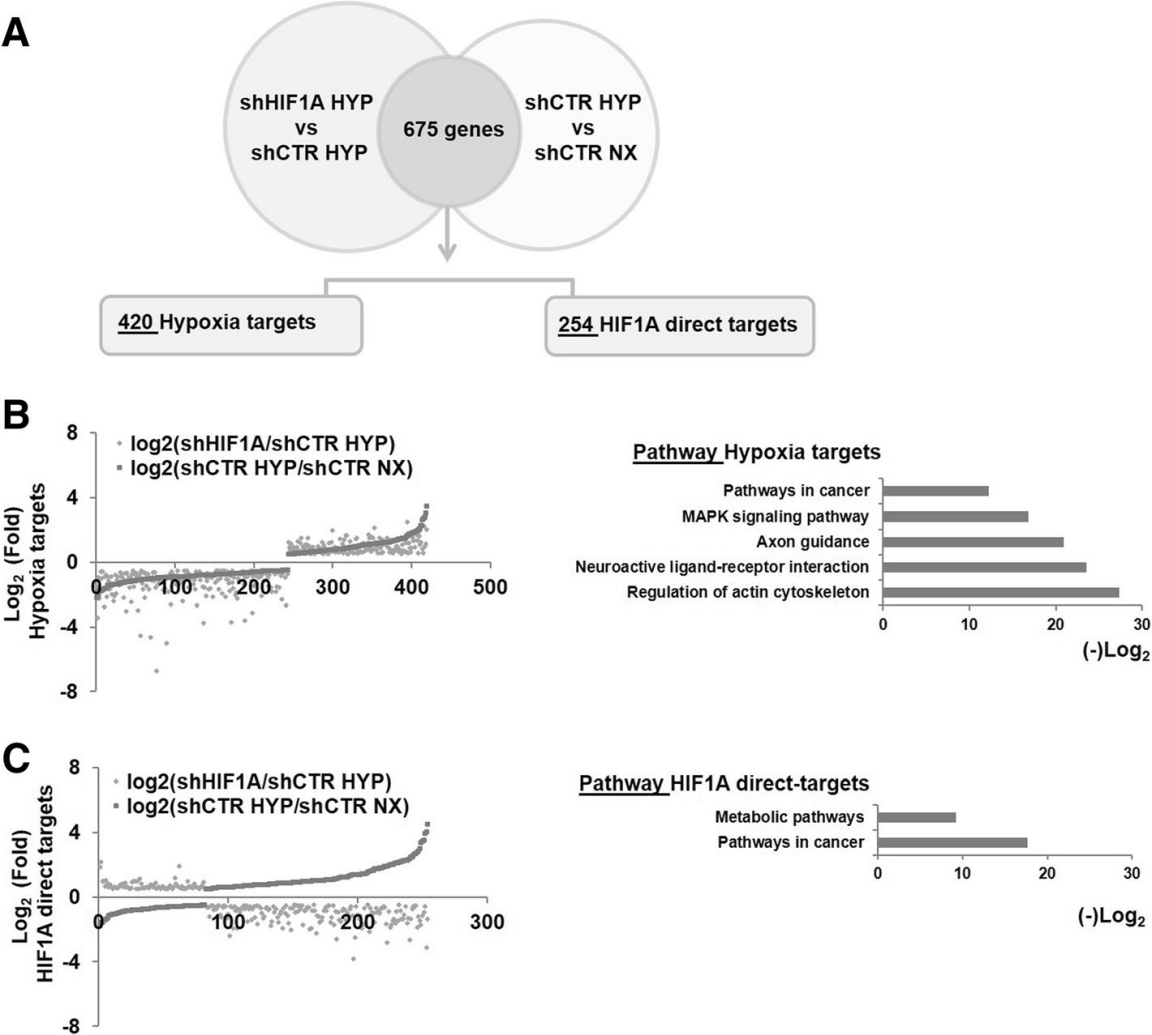
Genes regulated upon hypoxia exposure, in presence and absence of HIF1A. **a** The differentially expressed genes in shHIF1A HYP vs shCTR HYP and shCTR HYP vs shCTR NX were crossed and 674 genes were found commonly regulated. Of note, 420 out of 674 genes show the same trend of regulation and are named Hypoxia targets; 254 out of 674 genes show an opposite regulation and are named HIF1A targets. **b** The fold change of Hypoxia targets expression (Log_2)_ in both gene lists is shown in the graph; KEGG pathways analysis (*p*value ≤ 0.05) is reported and the negative Log_2_ pvalue is shown on X-axis. **c** The fold change of HIF1A direct targets expression (Log_2)_ in both gene lists is shown in the graph; KEGG pathways analysis (*p*value ≤ 0.05) is reported and the negative Log_2_ pvalue is shown on X-axis

The above results clearly highlight that HIF1A has a diverse role in normoxia than in hypoxia. Our hypothesis is that HIF1A driven response depends on the epigenetic reprogramming caused by low oxygen levels that may shape chromatin state and give HIF1A accessibility to HRE DNA regions previously closed. Furthermore, chromatin may remodel in regions not comprising HIF1A targets and allow HIF1A and other transcription factors access to new active DNA regions. To deeply investigate which genes are HIF1A dependently and HIF1A independently expressed, gene set shHIF1A HYP vs shCTR HYP (*n* = 1886 gene, Additional file 3: Table S2) and gene set of shCTR HYP vs shCTR NX (*n* = 3263 genes, Additional file 3: Table S3) were crossed. We found that 674 genes were regulated in both gene sets (Fig. 2a). Interestingly, 420 out of 674 genes show the same regulation in both gene sets (Log_2_); the expression of these genes named “Hypoxia targets” is affected by low oxygen concentrations and not affected by HIF1A (Fig. 2b). KEGG pathway analysis shows that “Hypoxia targets” are enriched in pathway that regulate cytoskeleton, ligand-receptor interaction and axon guidance (FDR ≤ 0.05). By contrast, 254 out of 674 genes have an opposite regulation in the two lists (Log_2_). These genes might be direct targets of HIF1A (here named: “HIF1A direct-targets”) in hypoxia because when HIF1A is depleted their regulation is inverted (Fig. 2c). KEGG pathway analysis shows that “HIF1A direct-targets” are enriched in metabolic and cancer pathways similar to pathways affected by HIF1A silencing “exclusively in hypoxia” (FDR ≤ 0.05). These findings suggest that NB cells adapt to hypoxia by HIF1A-dependent and HIF1A-independent driven response.

### DNA sites have variable methylation status under hypoxia exposure

**Fig. 3:**
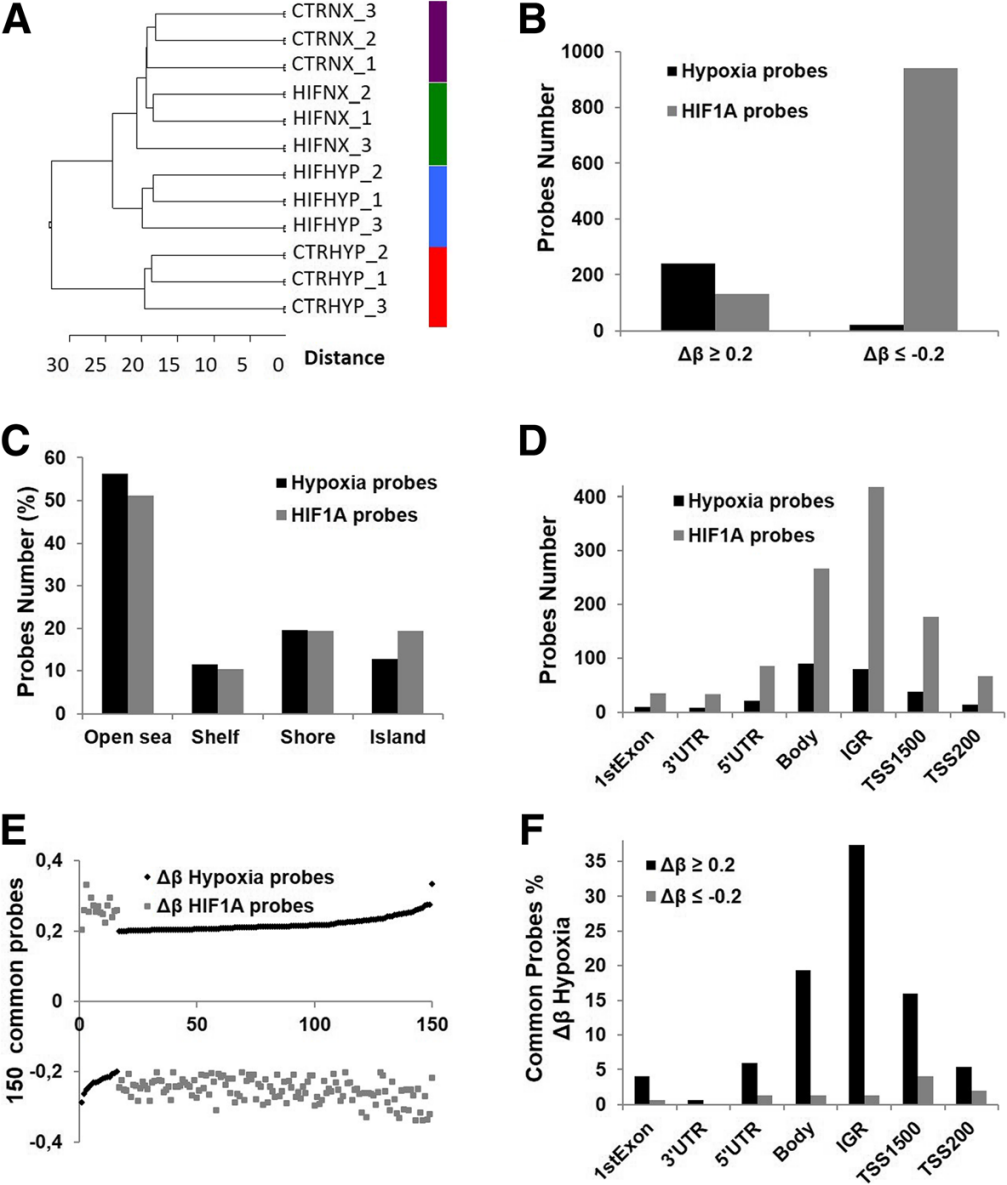
DNA sites with variable methylation under hypoxia. **a** The top 1000 most variable CpG probes were used to perform hierarchical clustering based on Euclidean distances. The analysis well separated samples into four clusters grouping all the replicates of each experimental condition. **b** Overall distribution of hyper (Δβ ≥ 0.2) and hypo-methylated (Δβ ≤ − 0.2) probes of “Hypoxia probes” (shCTR HYP vs shCTR NX). and “HIF1A probes” (shHIF1A HYP vs shCTR HYP) **c** Distribution of “Hypoxia probes” and “HIF1A probes” in relation to CpG-centric annotation. **d** Distribution of “Hypoxia probes” and “HIF1A probes” to Gene-centric annotation. **e** Plot showing the inverse methylation status (β values) of 150 probes (common probes) commonly methylated in “Hypoxia probes” and “HIF1A probes”. **f** Gene-centric distribution of hyper (Δβ ≥ 0.2) and hypo-methylated (Δβ ≤ − 0.2) common probes

Genome-wide methylation analysis using Infinium HumanMethylation450 BeadChips was performed in triplicate as described for RNAseq. To get an overview of the methylation patterns in the normalized data, hierarchical clustering of the most variable probes was performed. The analysis separated samples into four clusters, one for each experimental condition, within which replicates are grouped (Fig. 3a). Probes hypo or hyper-methylated with a Δβ-value greater than 0.2 (20%) in shHIF1A HYP vs shCTR HYP (named HIF1A probes) and in shCTR HYP vs shCTR NX (named Hypoxia probes) were selected. The sets of HIF1A probes and Hypoxia probes include 1078 (Additional file 3: Table S4) and 260 (Additional file 3: Table S5) differentially methylated CpG sites respectively. A global hypermethylation status of Hypoxia probes (Δβ ≥ 0.2) and hypomethylation status of HIF1A probes (Δβ ≤ − 0.2) was observed (Fig. 3b). Both probe sets cluster close to each other (Fig. 3a) whereas probes differentially methylated in shHIF1A NX vs shCTR NX were not observed.

We measured the genomic distribution of Hypoxia probes and HIF1A probes in relation to CGI centric annotation. Hypoxia probes were overrepresented in regions of low CpGs (open sea, > 4Kb from the CGI) and underrepresented in CGIs (island). Once HIF1A is depleted (HIF1A probes) open sea and island regions became under- and over-represented, respectively, (Fig. 3c). CGI shelves (>2Kb from the CGI) and shores (<2Kb from the CGI) showed a non-random distribution between the two probe sets. Hypoxia probes and HIF1A probes were mapped in relation to gene positions. In both contrasts, a strong overrepresentation of probes in intergenic regions (IGR) and gene body as well as an overall underrepresentation of probes located in the first exon, 3′ and 5’UTR and in the upstream region of transcription site (TSS) was observed (Fig. 3d). Hypoxia probes and HIF1A probes were crossed and 150 probes were found methylated in both probes lists. The methylation status (Δβ) of 150 common probes in hypoxia (shCTR HYP vs shCTR NX, Hypoxia probes) is reverted once HIF1A is depleted (shHIF1A HYP vs shCTR HYP, HIF1A probes) (Fig. 3e) and a strong overrepresentation of common probes in IGR is observed (Fig. 3f). Overall, these results suggest DNA methylation status is strictly correlated with oxygen storage and HIF1A control of DNA methylation of IGR, gene body and TSS probes could occur only upon hypoxia induced epigenetic reprogramming.

### Correlation of DNA methylation and gene expression under hypoxia exposure

**Fig. 4:**
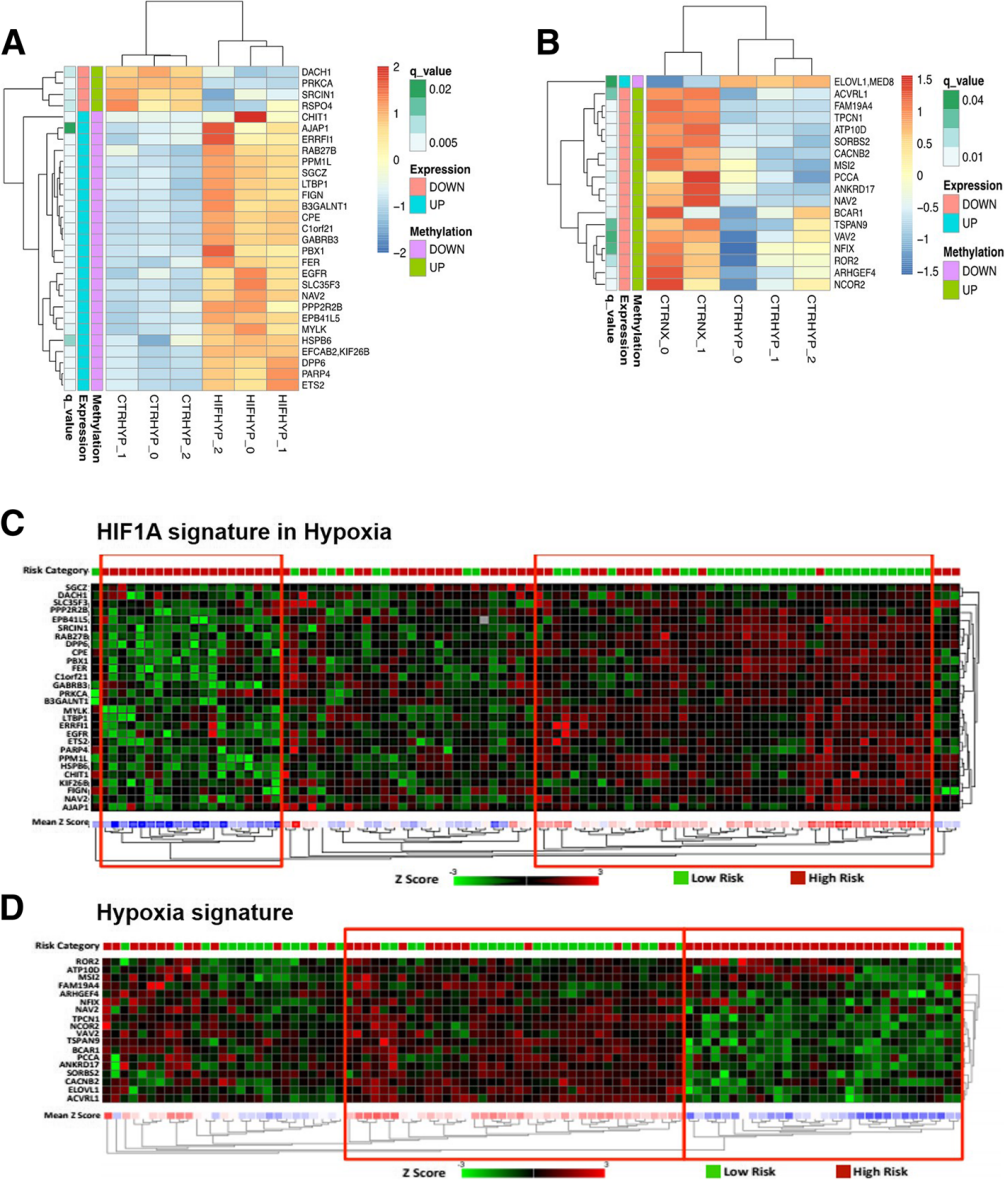
Correlation of DNA methylation and gene expression in NB cells and in NB samples. Gene expression heat maps showing the correlation of gene expression with the methylation status (annotation tracks on the left) in shHIF1A HYP vs shCTR HYP (**a**) and in shCTR HYP vs shCTR NX (**b**) pairwise contrasts. “q-value”: FDR. **a** and **b** Obtained with the R package “Pheatmap”. Gene expression heat maps show genes included in **c** “HIF1A signature in hypoxia” and **d** “Hypoxia signature”. Low risk (*n* = 40; in green) and High risk (*n* = 56; in red) patients of the GSE73517 data set were used to draw the heat maps.. Red boxes indicate clusters of Low risk and High risk samples. **c** and **d** Obtained on the R2: Genomics Analysis and Visualization Platform (http://r2.amc.nl). **a-d** The expression values were first Z-Score transformed and then used to perform hierarchical clustering based on Euclidean distances. Z-Score ranges are reported in the color keys

The correlation between the differential expression and differential methylation was explored for each gene-probe pair (*p*-value ≤0.05). We searched for changes in opposite directions (eg. Up-regulation of the gene expression and hypo-methylation of the related CpG probe). In the shHIF1A HYP vs shCTR HYP comparison, we selected 31 gene-probe pairs (*p* ≤ 0.05, Fig. 4a, Table 1), whereas in the shCTR HYP vs shCTR NX comparison 18 gene-probe pairs were kept (p ≤ 0.05, Fig. 4b, Table 1). These probes are located on regulatory regions as UTR, TSS200, TSS1500 and in gene bodies (Additional file 2: Figure S5).

**Fig. 5:**
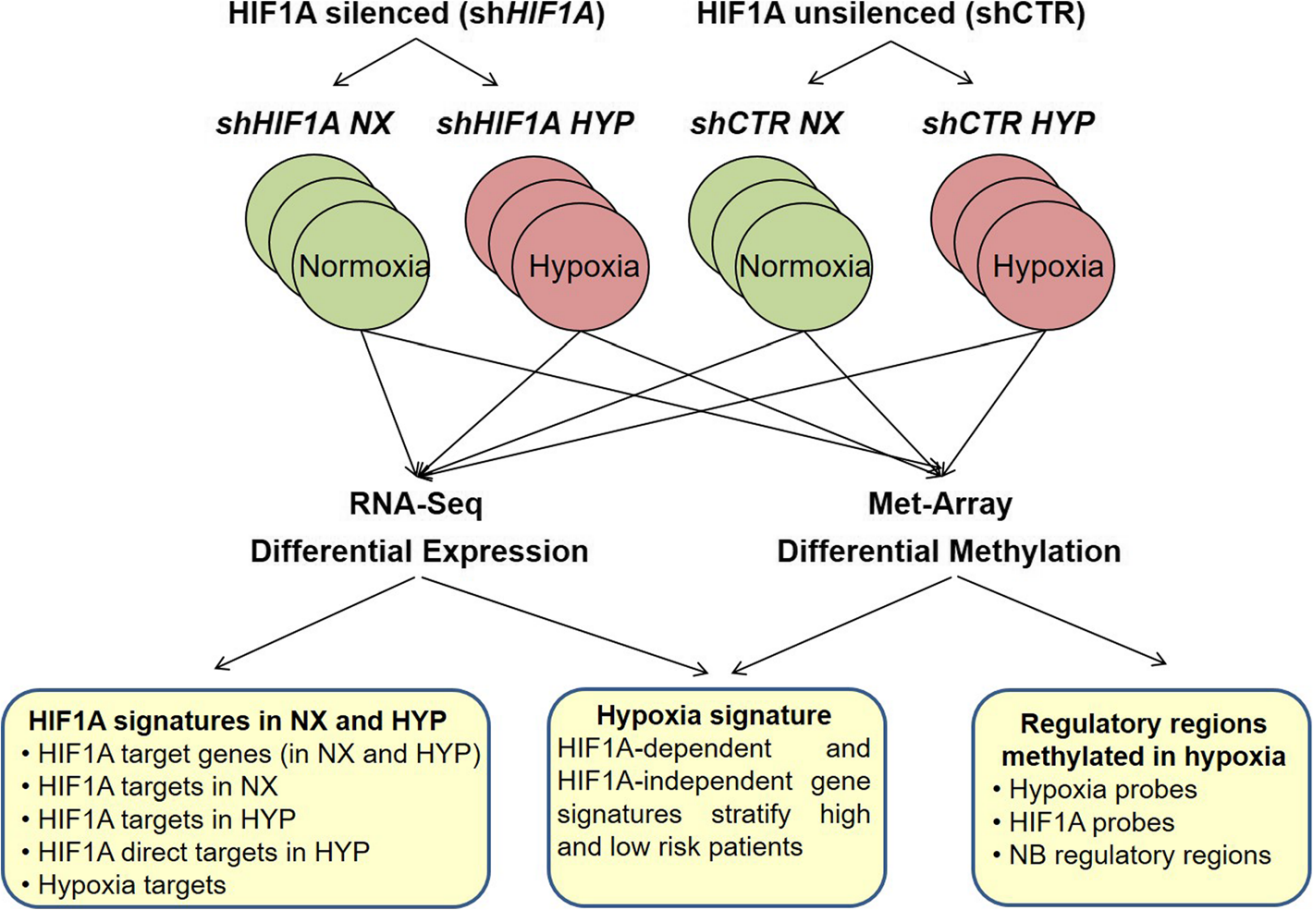
Transcription activity of HIF1A in normoxia and hypoxia conditions. To investigate the transcription activity of HIF1A in normoxia and hypoxia conditions RNA sequencing and DNA methylation experiments were performed on NB cells silenced or unsilenced for HIF1A expression. Experimental points were used in triplicate. Analysis of gene signatures and hypoxia affected regulatory regions was performed as sketched

**Table 1:** Correlation between DNA methylation (Probe ID) and gene expression (Gene) in hypoxia

ProbeID	Gene	Δβ HIF1A	Log2 FC HIF1A	Gene feature	CpG feature
cg01915791	HSPB6	− 0.24969157	0.773186	Body	island
cg02836965	SGCZ	−0.21032015	1.47509	Body	open sea
cg03387092	MYLK	−0.22225645	0.588606	Body	open sea
cg03905369	EPB41L5	−0.22872718	1.00335	Body	open sea
cg04056576	PPM1L	−0.22056766	0.857675	Body	shore
cg04194674	SRCIN1	0.21094717	−0.865002	Body	island
cg06221087	PBX1	−0.20339421	0.535856	Body	open sea
cg08843859	C1orf21	−0.23049642	0.924679	Body	open sea
cg11849717	EGFR	− 0.22631862	0.694851	Body	island
cg12743970	PRKCA	0.22709241	−0.728741	Body	open sea
cg14862207	SRCIN1	0.20762646	−0.865002	Body	island
cg16198315	DACH1	0.21650226	−0.533886	Body	shore
cg18277497	FIGN	−0.27273159	0.851071	Body	open sea
cg20897616	GABRB3	−0.24454897	1.42689	Body	island
cg21516044	CPE	−0.21441006	0.836739	Body	shelf
cg22340526	DPP6	−0.22509589	0.559082	Body	shore
cg24597512	GABRB3	−0.21252389	1.42689	Body	shore
cg24673955	KIF26B	−0.20727528	0.682105	Body	shore
cg25005674	PPP2R2B	−0.24873958	0.895844	Body	open sea
cg26672287	LTBP1	−0.22576329	0.82036	Body	open sea
cg27262041	NAV2	−0.21068975	0.518347	Body	open sea
cg27637738	EGFR	−0.2302424	0.694851	Body	open sea
cg21812277	PARP4	−0.21286384	0.619207	5’UTR	shore
cg08991927	PPP2R2B	−0.21036441	0.895844	5’UTR	shore
cg03690837	ETS2	−0.20969341	0.534019	TSS1500	island
cg11426075	ERRFI1	−0.21603014	0.555979	TSS1500	shore
cg13495205	AJAP1	−0.21926813	0.818246	TSS1500	island
cg18115428	SLC35F3	−0.2429891	1.76376	TSS1500	shore
cg24533917	CHIT1	−0.20768064	2.62046	TSS1500	open sea
cg26515460	RSPO4	0.21822875	−1.12443	TSS1500	shore
cg09565404	FER	−0.23534751	0.579076	TSS200	shore
cg13072057	B3GALNT1	−0.21923622	0.54531	TSS200	island
cg14135988	RAB27B	−0.20442834	0.713729	TSS200	island
cg14744537	RAB27B	−0.20487652	0.713729	TSS200	island
ProbeID	Gene	Δβ Hypoxia	Log2 FC Hypoxia	Gene feature	CpG feature
cg05502283	NFIX	0.22139292	−0.340491	3’UTR	shelf
cg18834544	PCCA	0.20607751	−0.553331	3’UTR	shore
cg12793733	ATP10D	0.2327763	−0.825063	5’UTR	open sea
cg15524063	ELOVL1	−0.21138562	0.340798	5’UTR	island
cg15883603	SORBS2	0.28146882	−1.01339	5’UTR	open sea
cg20164964	TSPAN9	0.21253777	−0.411039	5’UTR	open sea
cg22871175	FAM19A4	0.20371924	−0.80565	5’UTR	open sea
cg05490591	MSI2	0.21667094	−0.7364	Body	open sea
cg07238439	TPCN1	0.21883677	−0.778461	Body	open sea
cg11197258	NCOR2	0.2523067	−0.583474	Body	open sea
cg13764850	ROR2	0.21935471	−0.372244	Body	shelf
cg14377416	VAV2	0.21872117	−0.347793	Body	open sea
cg15570035	ANKRD17	0.20105082	−0.459873	Body	open sea
cg22218512	ACVRL1	0.20924927	−0.735922	Body	shore
cg26803268	CACNB2	0.2033665	−0.661703	Body	open sea
cg27262041	NAV2	0.21727999	−0.666122	Body	open sea
cg06888900	BCAR1	0.20187808	−0.642616	TSS1500	shore
cg07815799	ARHGEF4	0.27465952	−0.577694	TSS200	island

Δβ Hypoxia: Delta beta in shCTR HYP vs shCTR NX.; Δβ HIF1A: Delta beta in shHIF1A HYP vs shCTR HYP; Log2 FC: log2 of expression fold change

We explored gene-probe pairs correlation in 105 NB tumors for which matched methylation and gene expression data were available (GEO accessions: GSE73515 and GSE73517, respectively) and restricted our analysis to Low risk (*n* = 40) and High risk (*n* = 56) tumors as defined by Henrich et al. [28]. We found that the correlations for KIF26B, EFCAB2, BCL2L11, VAV2 and SORBS2 gene expression with methylation status were validated (Additional file 2: Figure S6A-E; *P* < 0.05). Additionally, in an independent set of NB tumors (GSE16476), we found that the expression of these genes was also associated to NB patient’s survival (Additional file 2: Figure S6F).

The two gene signatures generated from gene-probe pairs were named “HIF1A signature in hypoxia” and “Hypoxia signature”. To assess the prognostic potential of these signatures, we used Low risk (n = 40) and High risk (n = 56) tumors from the GSE73517 gene expression data set [28]. Hierarchical clustering based on Euclidean distances of expression levels, showed that the genes in “HIF1A signature in hypoxia” (Fig. 4c) clustered the 35.7% (20/56) of High risk and the 77.5% (31/40) of Low risk patients, in two separate groups (*P* < 1.0 × 10^− 4^) according to their Risk category. In contrast, “Hypoxia signature” (Fig. 4d), clustered the 50% (28/56) of High risk and the 57.5% (23/40) of Low risk patients according to their Risk category (P < 1.0 × 10^− 4^). Indeed, we verified that our gene signatures correctly classified a discrete portion of both High and Low risk patients.

### Identification of enhancers methylated under low oxygen conditions

The strong enrichment of hypoxia differentially methylated sites in IGR suggests that the changes of methylation pattern mainly occur at putative regulatory regions distant from target genes (as shown in Fig. 3d-f). To further select which hypoxia differentially methylated sites (Hypoxia probes *n* = 260) are located in NB putative regulatory regions we re-analysed DNase hypersensitivity assay and Chip-Seq histone acetylation (H3K27ac) data deposited in Gene Expression Omnibus database (GSE65664, Additional file 3: Table S6) for additional SKNBE2, CHP134 and SHSY5Y cell lines. We obtained that 113 out of 260 probes (43.5%) were located in regulatory active regions and 14 probes were annotated in all cell lines with at least 4 epigenetic markers (Additional file 3: Table S6). We searched the genes distant 1 Mb up- or down-stream from the 14 aforementioned probes in the RNAseq data (shCTR HYP vs shCTR NX, Log_2_ ≥ 0.2, ≤ − 0.2) and we listed the candidate targets of these regulative regions in Table 2 (and Additional file 3: Table S7). We observed that the methylation status of 9 out of 14 probes is affected by oxygen levels (Δβ Hypoxia; Hypoxia probes) but not by HIF1A expression (Δβ HIF1A; HIF1A probes). Putative targets show gene expression levels affected by oxygen levels (Log FC Hypoxia, in shCTR HYP vs shCTR NX, Log_2_ ≥ 0.2, ≤ − 0.2) and not by HIF1A expression (Log FC HIF1A, in shHIF1A HYP vs shCTR HYP, Log_2_ ≥ 0.2, ≤ − 0.2). Conversely, the methylation status of the remaining 5 probes is inversely affected in both conditions (Δβ Hypoxia and Δβ HIF1A) as the expression of putative targets (Log FC Hypoxia and Log FC HIF1A). The best NB candidate targets are represented by genes (highlighted in Table 2) whose expression correlates with NB patient’s event-free survival (Additional file 2: Figure S7).

**Table 2:**
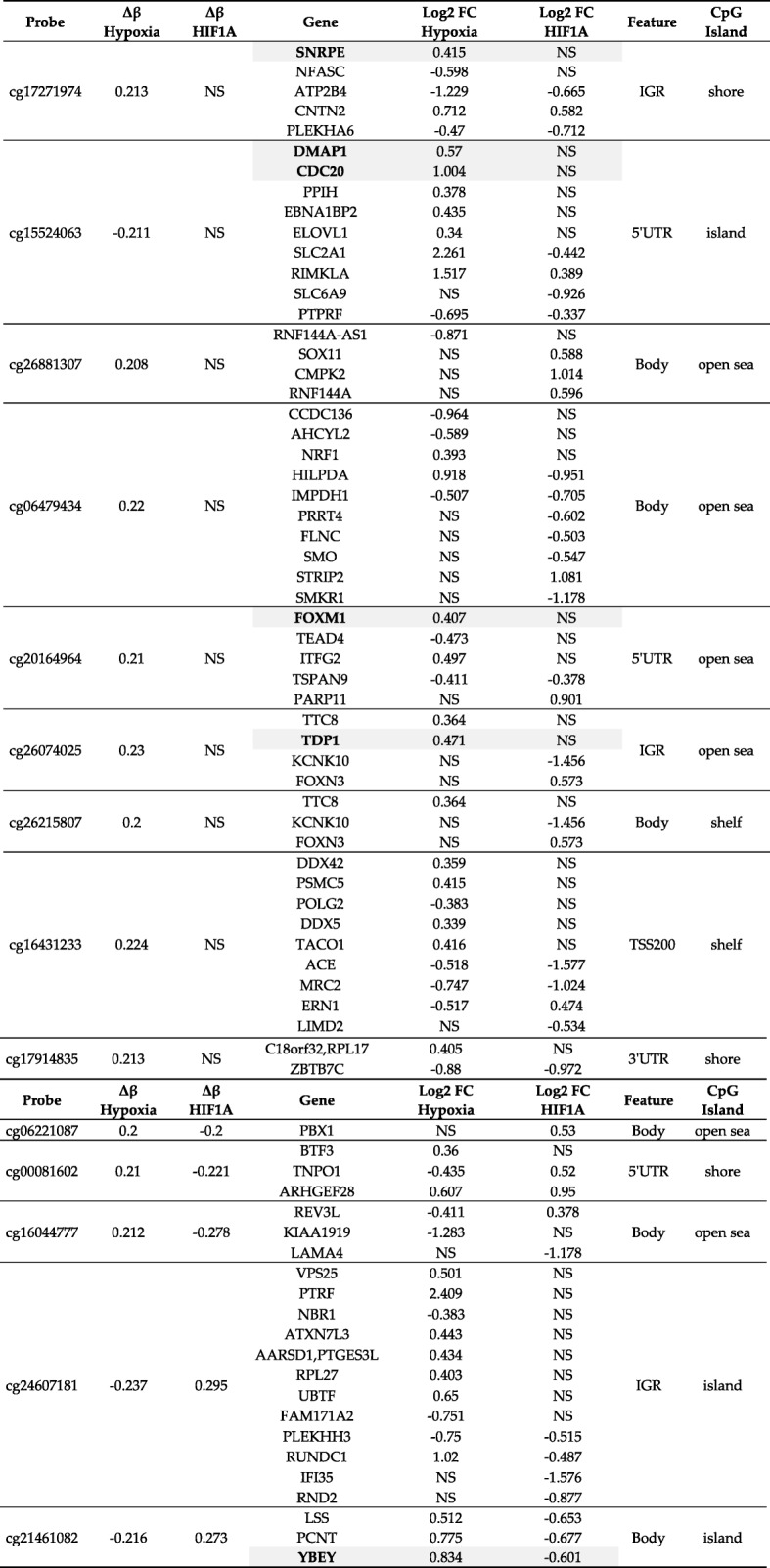
List of putative regulatory regions differentially methylated in hypoxia

Probes: putative enhancers; Δβ Hypoxia: Delta beta in shCTR HYP vs shCTR NX

Δβ HIF1A: Delta beta in shHIF1A HYP vs shCTR HYP. Log2 FC: log2 of expression fold change

Genes highlighted: best candidate targets

Abbreviations: *NS* Not significant

## Discussion

Increased expression of HIF1A in tumors is relevant to establish resistance to therapy [10, 11]. Interestingly, we have previously reported that high HIF1A expression may stratify high-risk NB patients with poorer prognosis [12].

Currently, targeting of hypoxia signaling has limitations in clinics with regard to changeable oxygen concentrations in solid tumor areas and HIF1A direct compounds do not show clinical efficiency. Indeed, the identification of HIF1A target genes and deep insights into the mechanisms of HIF1A driven gene expression may provide novel risk factors to meliorate survival/therapeutic successes in patients with high-risk tumors that lack of precisely genomic causes.

In the present study, we have investigated HIF-1 driven transcription activity in both hypoxic and normoxic conditions in NB cells depleted of HIF1A expression. The analysis of pathways regulated by HIF1A exclusively in normoxic NB cells shows a role of HIF1A in metabolic process necessary for tumor cells viability. Particularly, the global down-regulation of gene expression in absence of HIF1A suggests that NB cells slow down their metabolic activity, thus becoming less proliferating. HIF1A involvement in basic cellular activity, like glycolytic pathways, has been described [29].

Contrary, in hypoxic cells the absence of HIF1A affects the activation of neuronal differentiation pathways in line with literature data showing that low oxygen in environments causes de-differentiation of NB cells towards an immature and neural-crest-like phenotype [30]. We have previously highlighted HIF1A involvement in NB neuronal differentiation pathways activation and response to differentiating agents [12].

Interesting to note, mostly of genes regulated by HIF1A in both normoxic and hypoxic areas belong to MAPK pathways. This pathway is frequently altered in high-risk NB at relapse and at diagnosis and multiple drugs aimed to target MAPK signaling are used in current clinical trials for the treatment of metastatic tumors [5, 8, 31]. Indeed, HIF1A target genes in both normoxic and hypoxic areas may provide potential targets for a precision therapy. HIF1A is not the unique player to define the whole picture of hypoxia-regulated gene expression. In effect, we report that NB cells adapt to hypoxia by HIF1A-dependend and HIF1A-indipendent driven response. These findings help us to understand how oxygen is sensed at NB cellular levels.

We assume that HIF1A driven transcriptional response in hypoxia is a consequence of the epigenetic control of low oxygen levels at DNA methylation status. We have observed that hypoxia exposure induces a global DNA hypermethylation in NB cells and HIF1A itself might control DNA methylation status. A global DNA hypermethylation has been previously linked to poor NB prognosis as site-specific DNA hypermethylation of tumor suppressor genes to optimize the environment for cancer initiation and progression [32, 33]. The hypoxia epigenetic controls at the levels of RNA and proteins still remain to be explored.

Despite the stereotype, DNA methylation does not appear to play a major role in gene regulation from 5’CGI promoters of most genes in hypoxia. Indeed, few genes show a correlation between expression and methylation status of close regulatory regions and some correlations were validated in NB samples. Hypoxic gene signatures generated from this correlation analysis are able to stratify NB patients in two risk categories. Although numerous prognostic gene signatures have been developed to classify NB patients, none has been introduced into clinical risk stratification systems [2, 34, 35]. To overcome these limitations, the establishment of gene signatures that take into account the effects of oxygen levels in tumor bulk more than clinical and genetic markers may be an innovative strategy for NB stratification at diagnosis. Of course, these findings need independent validations.

Conversely, low oxygen levels and HIF1A affect the methylation status of probes located in intragenic and intergenic regions [36–38]. Most probes are located in NB active regulatory regions and the different methylation status correlates to different expression of distant candidate targets associated with NB survival. These genes have been previously associated to therapy resistance and cancer progression and may represent potential markers for NB.

CDC20 is a component of the mammalian cell-cycle mechanism and activates the anaphase-promoting complex (APC); its inhibition may enhance radio sensitivity in nasopharyngeal carcinoma cells [39].

SNRPE (small nuclear ribonucleoprotein polypeptide E) has oncogenic effects in prostate cancer [40]. TDP1 (Tyrosyl-DNA Phosphodiesterase 1) is DNA repair enzyme potential therapeutic target for the treatment of colorectal cancer [41].

FOXM1 (Forkhead Box M1) transcription factor regulates the expression of cell cycle genes and plays an important role in NB tumorigenicity through maintenance of cells undifferentiated state [42]. Interestingly, FOXM1 overexpression in hypoxia has been already documented in cancer [43].

DMAP1 (DNA Methyltrasferase 1 Associated Protein 1) contributes to epatocarcinoma malignancy [41].

YBEY (C21orf57) is a highly conserved metalloprotein not-well characterized in cancer.

High-throughput sequencing-based studies have shown low mutations frequency in coding-portion of NB genome and high recurrence of structural rearrangement. Previous genome-wide association studies revealed that many loci associated with NB susceptibility lie in non-coding regions of the genome [35, 44–46]. Based on these evidences, it is reasonable to expect that recurrent non-coding somatic mutations could have a regulatory effect in NB tumorigenesis. In light of all this, our results further underline the role of non-coding regulatory elements in driving NB tumorigenesis through epigenetic regulation in hypoxia. How epigenetic landscape in hypoxia contributes to transformations and how these alterations complement other acquired somatic mutations need to be elucidated.

One limitation of this study is the use of established cell lines that reflects limited aspects of in vivo tumor microenvironments. It lacks geometrical complexity, cellular components including immune cells and organ-specific stromal cells, and extracellular matrix components. Here, our aim was to establish a HIF1A-based method useful in the investigation of undiscovered mechanisms of neuroblastoma tumorigenesis under hypoxic microenvironments. However, our results still need to be confirmed by functional validation and mechanistic studies which could further improve in vitro cell line models predictive validity.

## Conclusions

Recent evidences of HIF1A gene expression increment in solid tumors and the stabilization of low HIF1A protein levels in normoxic cells highlight HIF1A transcription activity in both normoxic and hypoxic conditions. In the present study, we have investigated HIF1A targets activated in both oxygen level conditions by an analysis of gene expression and DNA methylation of NB cells silenced or unsilenced for HIF1A expression. We have verified that HIF1A transcription activity depends on oxygen levels and HIF1A targets regulated in both conditions might provide potential therapeutic targets to eradicate solid tumors. Hypoxia signatures might provide novel risk factors for NB stratification at diagnosis. Hypoxia regulates gene expression through an epigenetic control on regulatory elements distant from target genes (Fig. 5). Overall, the presented results may help to understand the molecular mechanisms by which hypoxia reshapes tumors and provide new direction for hypoxia solid tumor treatment.

## Additional files

Additional file 1: Supplementary Material and Methods cDNA library construction; Analysis of differentially expressed genes; Correlation of DNA methylation and gene expression; Primers sequences for RT-PCR. (DOC 55 kb)

Additional file 2: **Figure S1.** Main features of RNA-Seq data; **Figure S2.** Differentially expressed genes in shHIF1A NX vs shCTR NX and in shHIF1A HYP vs shCTR HYP gene sets; **Figure S3.** Validation of RNAseq data by RT-PCR in SHSY5Y cells; **Figure S4.** Correlation of DNA methylation and gene expression; **Figure S5.** Genes differentially expressed and methylated under hypoxia and associated with NB survival; **Figure S6.** Survival analysis of NB patients based on the gene expression of candidate targets of putative enhancers differentially regulated in hypoxia. **Figure S7.** Survival analysis of NB patients based on the gene expression of candidate targets of putative enhancers differentially regulated in hypoxia. (DOCX 7156 kb)

Additional file 3: **Table S1.** shHIF1A NX vs shCTR NX; **Table S2.** shHIF1A HYP vs shCTR HYP; **Table S3.** shCTR HYP vs shCTR NX; **Table S4.** Probes shCTR HYP vs shHIF1A HYP; **Table S5.** Probes shCTR HYP vs shCTR NX; **Table S6.** NB annotated enhancers; **Table S7.** Genes surrounding 14 probes. (XLS 1732 kb)

## Abbreviations

HIF-1: Heterodimeric transcription factor hypoxia-inducible factor 1
HIF1A: Hypoxia-inducible factor α
HYP: Hypoxia
NB: Neuroblastoma
NX: Normoxia
