# Supplementary material for: HIF-1 transcription activity: HIF1A driven response in normoxia and in hypoxia
Source: BMC Med Genet. 2019 Feb 26;20:37. doi: 10.1186/s12881-019-0767-1 (PMC6390360; doi:10.1186/s12881-019-0767-1)
Supplement: Supplementary file 1 — Supplementary Material and Methods cDNA library construction; Analysis of differentially expressed genes; Correlation of DNA methylation and gene expression; Primers sequences for RT-PCR. (DOC 55 kb) [file 12881_2019_767_MOESM1_ESM.doc]

**Supplementary Material and Methods**

**cDNA library construction**

Total RNA samples quality was assessed with Agilent 2100 Bioanalyzer RNA Nano chip device (Agilent, Santa Clara, CA, USA), and total RNA concentrations were determined using a NanoDrop ND1000 spectrophotometer (Nano-Drop, Wilmington, DE, USA). RNA with an OD260/280 between 1.8 and 2.2 and an OD260/230 ≥ 1.8 was used for the construction of cDNA libraries. The mRNA content was concentrated from total RNA using RNase-free DNase I (TaKaRa) and magnetic oligo (dT) beads. The mRNA was mixed with the fragmentation buffer and broken into short fragments (~200 bp long). Then, the first strand of cDNA was synthesized with random hexamer primers. The second strand was synthesized using the SuperScript Double-Stranded cDNA Synthesis kit (Invitrogen, Camarillo, CA) and was purified via magnetic beads. The ends were repaired and tailed with a single 3’ adenosine. Subsequently, the cDNA fragments were ligated to sequencing adapters.

**Analysis of differentially expressed genes**

The analysis started from cleaned data previously filtered by adapter sequences, low quality reads and from reads with more than 10% of unknown bases. Quality assessment on cleaned FASTQ files was performed using FastQC (<http://www.bioinformatics.babraham.ac.uk/projects/fastqc>).

Sequencing data were analysed with the set of open source software programs of the Tuxedo suite: TopHat v2.0.14 and Cufflinks v2.1.0, following the analysis pipeline published in nature protocols 2012 .^21^ TopHat is a fast splice junction mapper for RNA-Seq reads. It aligns RNA-Seq reads to the reference genome using the ultra high-throughput short read aligner Bowtie2 v2.2.6.0, and then analyzes the mapping results to identify splice junctions between exons. TopHat was ran with default options by providing the reference genome (assembly GRCh37/hg19 downloaded from UCSC and indexed with Bowtie2) and its related RefSeq reference transcriptome (downloaded from UCSC) along with the couple of FASTQ files (forward and reverse reads) for each sample. The alignment versus the reference genome (GRCh37/hg19 assembly) had an overall mapping rate of 80%, as shown in the table below.

| **Sample** | **reads Fwd (left)** | **reads Rev (right)** | **overall mapping rate** | **pair alignment rate** |
| --- | --- | --- | --- | --- |
| CTRHYP_1 | 22533275 | 22533275 | 79.50% | 73.10% |
| CTRHYP_2 | 22162261 | 22162261 | 81.40% | 75.00% |
| CTRHYP_3 | 22406261 | 22406261 | 79.50% | 73.20% |
| CTRNX_1 | 15908308 | 15908308 | 70.80% | 64.60% |
| CTRNX_2 | 19756627 | 19756627 | 82.70% | 76.10% |
| CTRNX_3 | 19328342 | 19328342 | 83.00% | 76.30% |
| HIFHYP_1 | 17113354 | 17113354 | 81.30% | 74.60% |
| HIFHYP_2 | 19090920 | 19090920 | 78.40% | 71.40% |
| HIFHYP_3 | 18532728 | 18532728 | 77.50% | 70.70% |
| HIFNX_1 | 13497762 | 13497762 | 82.60% | 75.40% |
| HIFNX_2 | 23083550 | 23083550 | 79.00% | 72.50% |
| HIFNX_3 | 22993764 | 22993764 | 81.70% | 75.30% |
| **Median** | **19542484.5** | **19542484.5** | **80%** | **74%** |
| **Average** | **19700596** | **19700596** | **80%** | **73%** |

Next, Cufflinks was used to assemble the mapped reads (in the BAM file format) into possible transcripts and to generate a final transcriptome assembly. The tool Cuffmerge was exploited to merge transcriptomes from all samples and generate a common transcriptome file. Cuffdiff was finally used to detect differentially expressed genes and transcripts. It takes mapped reads from two or more biological conditions (provided as two or more biological replicates) and analyzes their differential expression of genes and transcripts, thus aiding in the investigation of their transcriptional and post transcriptional regulation under different conditions. The program was ran by providing all the obtained BAM files (specifying the experimental condition and the replicate to which they belonged), the merged transcriptome assembly and the sequence of the reference genome. The software returned: FPKM counts (Fragments Per Kilobase Of Exon Per Million Fragments Mapped) for each replicate, averaged FPKM counts for each experimental condition, and pairwise comparisons from the different experimental conditions (reporting the differences in expression levels). The set of output files obtained by Cuffdiff, reporting was then inspected and explored using the R-Bioconductor package CummeRbund v2.16.0, which provides functions to read, subset, filter and plot results.

**Correlation of DNA methylation and gene expression**

The correlation between the differential expression and differential methylation was explored for each gene-probe pair. In brief, for each contrast, we intersected the lists of significant differentially expressed genes (Log2 FC ≤ -0.5, down-regulated, or Log2 FC ≥ 0.5, up-regulated, and FDR ≤ 0.05) with the lists of significant differentially methylated probes (Δβ < -0.2, hypo-methylated or Δβ ≥ 0.2, hyper-methylated, and FDR < 0.05). Next, for each condition within a contrast we calculated gene-probe level T-test p values between expression and methylation status of 3 replicates and selected gene-probe pairs with p ≤ 0.05. Finally, we kept only those pairs that showed opposite trend between expression and methylation status in each contrast.

**Primers sequences for RT-PCR**

| **Gene** | **PRIMER FORWARD** | **PRIMER REVERSE** |
| --- | --- | --- |
| **CDH7** | GGACTGTCAGGAACTACATCAGTCA | ATAAGACCTTCGAGGAAAGCGA |
| **PCDH17** | GGGAGGCACTCAAGATGAAAAC | CTGTGCAATTAACACACTCTTCTGG |
| **DACH1** | TTCCATCTCCTTTTCTGTTTCCTG | CAACTTTCAACAGCCCCTGTATG |
| **LAMA4** | ACCGGAGAATGCTTGGAAGAA | CCAGACGCACTTATCACAGCTTAT |
| **SH3RF3** | CAACGTGTACCTGGCGCTCTA | ACCCGGTACATCTCTCCCTTG |
| **SPON1** | GGCTCTCTGACCAAGAAACTTTGT | GGCACAGCAGTCTAAGATGGGT |
| **TMEM45A** | AACCATTGTCATCGTTGGAATG | TCTGAGGAGCAGAGCCTCTTAAGT |
| **AJAP1** | ACATTTAATGGAAACCGACCCTC | GATTTCAAACCATTTCTCAGACACG |
| **GARB3** | TCTGGAAATTGAAAGCTATGGCTAC | ACTCCGGTAACAGCCTTGTCC |
| **KIF26B** | CGATGCAGTTTTTCCACAAGAC | GTTGACCACAGACTGGATCACCT |
| **PARP4** | TGTGGTGCCGGAGTATTTGAA | CCTGGTCATTTGGTCTTCTATCTGT |
| **SLC35F3** | AGTCCACAGAGAAGCAGTCTGTGA | CCTTCAAAGTCAAGCCATTGTCT |
| **NAV2** | ACTTGGCCTCTATACCCGTCG | GAGGTATTTCGTTGCAGGGTCT |
