# Supplementary material for: HIF-1 transcription activity: HIF1A driven response in normoxia and in hypoxia
Source: BMC Med Genet. 2019 Feb 26;20:37. doi: 10.1186/s12881-019-0767-1 (PMC6390360; doi:10.1186/s12881-019-0767-1)
Supplement: Supplementary file 2 — Figure S1. Main features of RNA-Seq data; Figure S2. Differentially expressed genes in shHIF1A NX vs shCTR NX and in shHIF1A HYP vs shCTR HYP gene sets; Figure S3. Validation of RNAseq data by RT-PCR in SHSY5Y cells; Figure S4. Correlation of DNA methylation and gene expression; Figure S5. Genes differentially expressed and methylated under hypoxia and associated with NB survival; Figure S6. Survival analysis of NB patients based on the gene expression of candidate targets of putative enhancers differentially regulated in hypoxia. Figure S7. Survival analysis of NB patients based on the gene expression of candidate targets of putative enhancers differentially regulated in hypoxia. (DOCX 7156 kb) [file 12881_2019_767_MOESM2_ESM.docx]

**Figure S1**

**A**

**
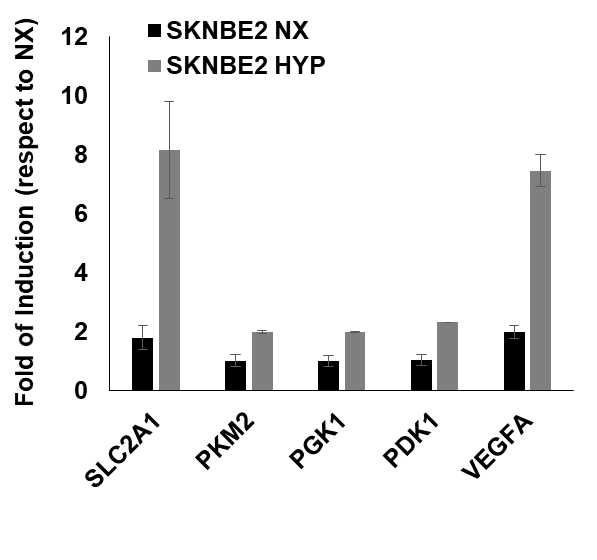
**

**Figure S1**. RT-PCR of hypoxia targets to evaluate hypoxia status. We tested by RT-PCR the gene expression of well-known targets of HIF1A and hypoxia. As shown in graph, hypoxia targets levels increase in SKNBE2 exposed to hypoxia (HYP), respect to normoxia cells (NX), and suggested that 2 hrs of incubation in low oxygen made the cells hypoxic. The data are fold-changes of induction with respect to NX cells (P ≤0.05).

The gene-specific primers were designed using the PRIMEREXPRESS software (Applied Biosystems), as: SLC2A1 (F): CCTGCTCATCAACCGCAAC; (R): TCATGGGTCACGTCAGCTGT; PKM2 (F): GCCATAATCGTCCTCACCAAGT; (R): TCCGGGTCACAGCAATGAT; PGK1 (F): CCAAGACTGGCCAAGCCA; (R): TCCTTGCTGCTTTCAGGACCAC; PDK1 (F): TCTCCATGAAGCAGTTCCTGG; (R): GCAACTCTTGCCCGCAGAAAC; VEGFA (F): CTACCTCCACCATGCCAAGT (R): GCAGTAGCTGCGCTGATAGA; β-Actin (F) CGTGCTGCTGACCGAGG; (R): GAAGGTCTCAAACATGATCTGGGT.

**Figure S2**

**Figure S2. Main features of RNA-Seq data**. Jensen-Shannon distances Dendrogram based on gene expression values of the different experimental conditions (**A**). Overall gene expression box plot (FPKM: Fragments per kilobase of exon per million reads mapped) (**B**). Principal Component Analysis (**C**) and Multi-dimensional scaling (**D**) showing the differences between different experimental condition datasets. The plots show the averaged data of 3 biological replicates per experimental condition.

**Figure S3**

**A**

**
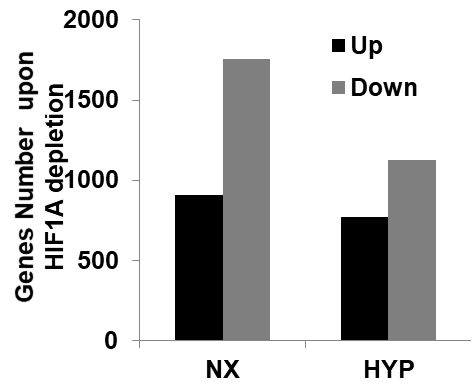
**

**B**

**
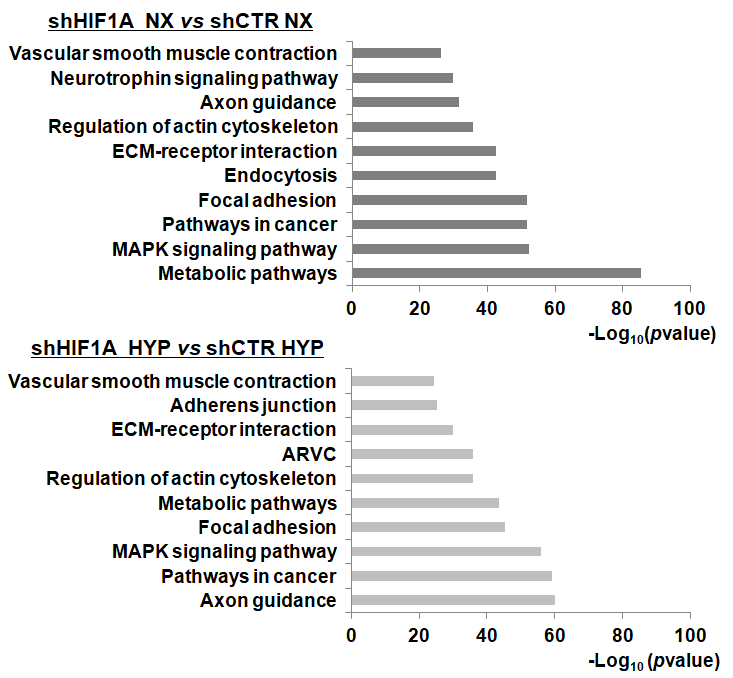
**

**Figure S3. Differentially expressed genes in shHIF1A NX vs shCTR NX and in shHIF1A HYP vs shCTR HYP gene sets.**The number of genes differentially expressed in both gene lists is shown in (A) KEGG pathways analysis of genes regulated upon HIF1A silencing in both oxygen conditions were performed by using the WEB-based GEne SeT AnaLysis Tool (WebGestalt) and showed that the most significantly enriched terms were metabolic pathway in normoxia and axon guidance in hypoxia. Pathway analysis results were filtered for *p*value ≤ 0.05 (FDR corrected). The negative Log_10_ pvalue is reported on X-axis (B).

**Figure S4**

The human SHSY5Y (ATCC #CRL-2266) cell line was grown in Dulbecco’s modified Eagle’s medium supplemented with 10% heat inactivated fetal bovine serum (Sigma), 1 mM L-glutamine, penicillin (100 U/ml) and streptomycin (100 μ g/ml) (Invitrogen), at 37 °C, under 5% CO2 in a humidified atmosphere. The cells exposed to hypoxia were grown at 0.5 % oxygen for 2 h. The cell line was reauthenticated and tested as mycoplasma-free. Early-passage cells were used and cumulative culture length was less than 3 months after resuscitation.

**A**

**
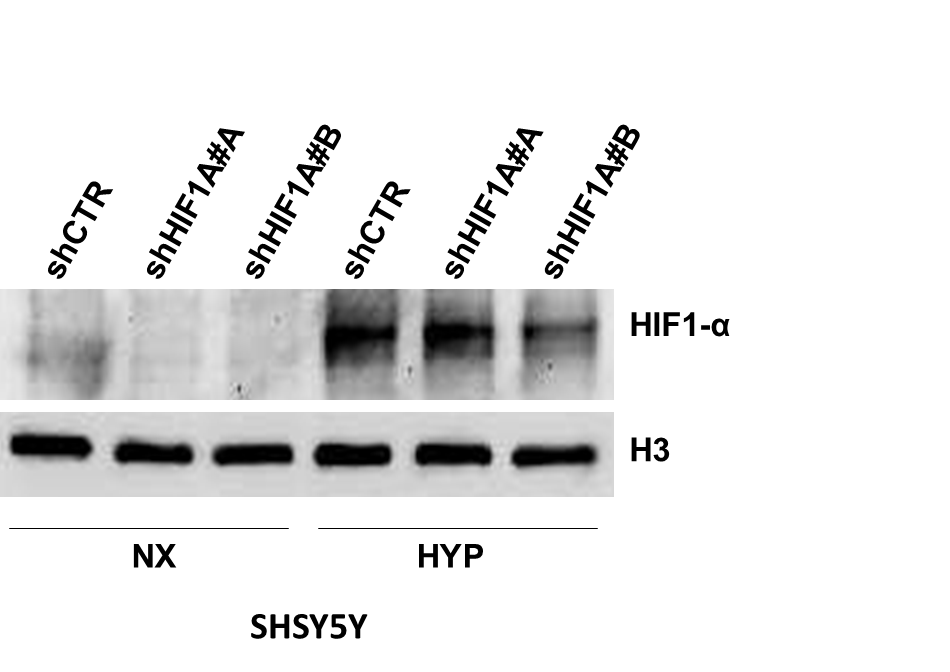
** **
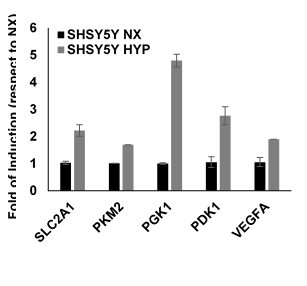
**

**B**

**
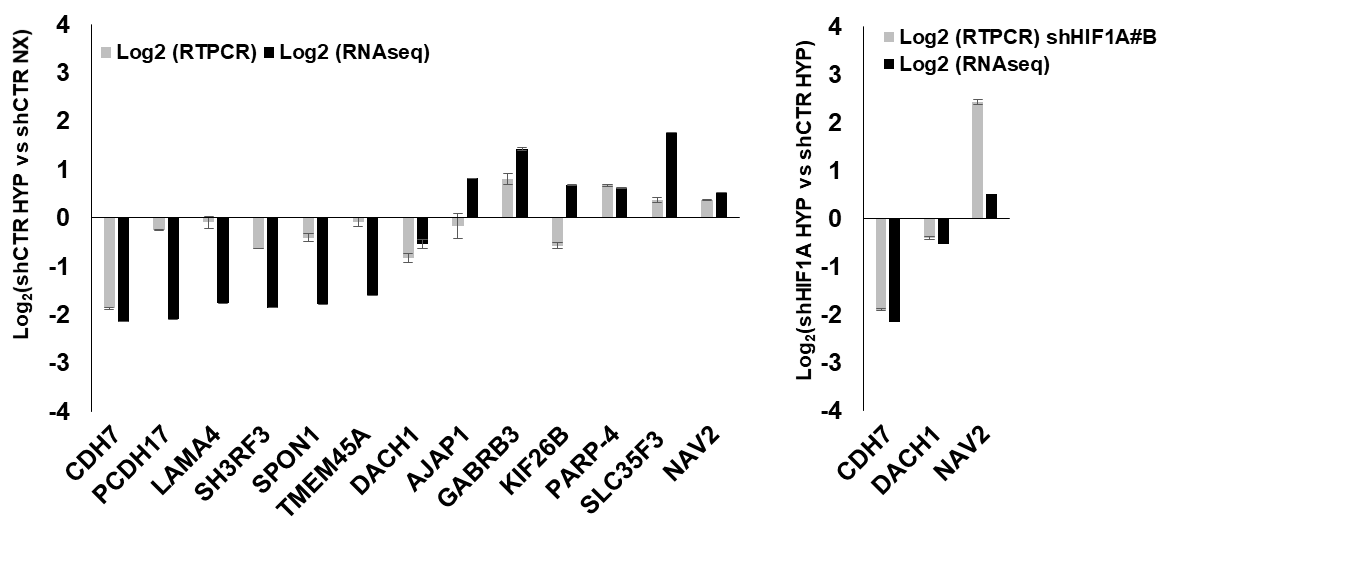
**

**Figure S4. Validation of RNAseq data by RT-PCR in SHSY5Y cells.** SHSY5Y cells silenced for HIF1A (shHIF1A) and unsilenced cells (shCTR) were grown in normoxia and hypoxia (0.5% O_2_, 2 hours). HIF-1α silencing efficiency was verified on nuclear extracts by western blotting and H3 has been used as loading control. RT-PCR to evaluate the expression levels of hypoxia target genes (SCL2A1, PKM2, PGK1, PDK1, VEFGA) was performed on shCTR NX and shCTR HYP cells. Gene expression of β-Actin was used as loading control. The data are fold-changes of induction with respect to the shCTR NX (P ≤0.05). (**A**). The reliability of RNAseq data was estimated by assessing the expression values of chosen genes by RT-PCR in SHSY5Y shCTR and shHIF1A#B cells. The chosen genes have RNAseq log_2_ fold change ≤ -2 and ≤ 2, in shCTR HYP vs shCTR NX gene list. The genes LAMA4, AJAP and TMEM45A do not show changes of expressions, KIF26B shows an opposite regulation. The expression values of three genes were also validated in shHIF1A (shHIF1A#B) HYP vs shCTR HYP (**B**).

**Figure S5**

**A**

**B**

**Figure S5. Correlation of DNA methylation and gene expression.** Distribution of differentially methylated probes associated with differentially gene expression in shHIF1A HYP vs shCTR HYP (**A**) and in shCTR HYP vs shCTR NX (**B**) pairwise comparisons. Gene-centric annotation is color-coded while bar labels are related to CpG island regions annotation.

**Figure S6**

**Figure S6. Genes differentially expressed and methylated under hypoxia and associated with NB survival.** Table reporting the correlation of the differential expression (Log2 FC) and differential methylation (deltaBeta) of Gene-Probe pairs between our data (this study) and a dataset of 105 NB tumors (Westermann, GEO accession numbers GSE73517 and GSE73515). P values for the High Risk vs Low Risk contrasts in the Westermann data were obtained with the T-test. Significant P values are shown in bold (**A**). We drawn boxplots for the significant Gene-Probe pairs correlations from above (**B-E**). The expression of these selected genes was also found associated to prognosis in an independent set of NB tumors (GSE16476, Versteeg dataset). All the analyses were performed on the 'R2: Genomics Analysis and Visualization Platform (<http://r2.amc.nl>) (**F**).

**Figure S7**


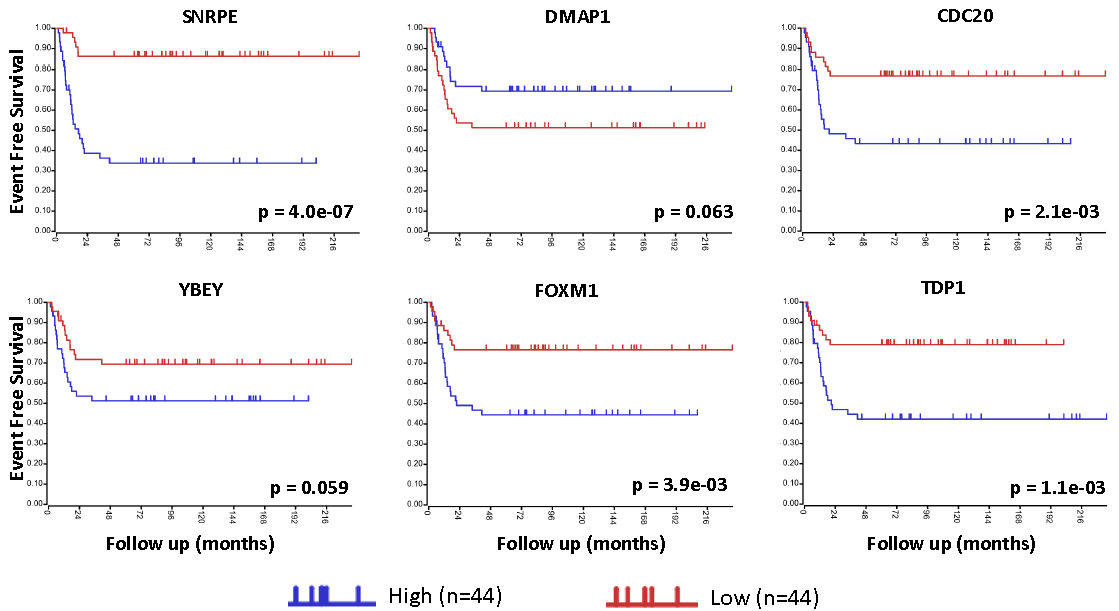


**Figure S7. Survival analysis of NB patients based on the gene expression of candidate targets of putative enhancers differentially regulated in hypoxia.** The correlation between gene expression and overall survival has been performed on a cohort of 88 NB patients (GSE16476, Versteeg dataset). Survival analysis was performed on the 'R2: Genomics Analysis and Visualization Platform (http://r2.amc.nl)'.
